# Oral Melanoacanthoma of a Rare Intraoral Site: Case Report and Review of Literature

**DOI:** 10.5005/jp-journals-10005-1185

**Published:** 2013-04-26

**Authors:** Kshitiz Rohilla, V Ramesh, PD Balamurali, Namrata Singh

**Affiliations:** Demonstrator, Department of Oral Pathology, Postgraduate Institute of Dental Sciences, Rohtak, Haryana, India; Dean, Professor and Head, Department of Oral Pathology and Microbiology, Mahatma Gandhi Postgraduate Institute of Dental Sciences, Puducherry, India; Professor, Department of Oral Pathology and Microbiology, Mahatma Gandhi Postgraduate Institute of Dental Sciences, Puducherry, India; Ex-Senior Lecturer, Department of Orthodontics and Dentofacial Orthopedics, Indira Gandhi Institute of Dental Sciences, Puducherry India

**Keywords:** Melanoacanthoma, Oral pigmented lesion, Melanocytes

## Abstract

Oral melanoacanthoma is rare pigmented mucosal lesion that presents most commonly on the buccal mucosa, characterized by sudden appearance and rapid radial growth, thus clinically mimicking malignant melanoma. It was originally described as a mixed tumor of melanocytes and keratinocytes, but appears to be a reactive process; formed in areas prone to trauma, and regressing after the removal of trauma or incomplete excision. The clinical appearance of oral melanoacanthoma is nondiagnostic, and biopsy is mandatory to rule out malignancy. We report a case of melanoacanthoma of a rarer oral mucosal site in a 12-year-old Asian male. A brief review of the current literature is also presented.

**How to cite this article:** Rohilla K, Ramesh V, Balamurali PD, Singh N. Oral Melanoacanthoma of a Rare Intraoral Site: Case Report and Review of Literature. Int J Clin Pediatr Dent 2013;6(1):40-43.

## INTRODUCTION

Melanoacanthoma is an uncommon, benign, mucocutaneous pigmented lesion characterized by dendritic melanocytes dispersed throughout the acanthotic epithelium.^1,2^ Though it was originally described as a benign skin tumor of keratinocytes and dendritic melanocytes, there is now evidence that the intraoral lesions are unlike those occurring on skin.^3^

Cutaneous melanoacanthoma was first described in 1927 by Bloch, but the term melanoacanthoma was introduced by Mishima and Pinkus in 1960.^1^ The first case of oral melanoacanthoma was reported in 1978 by Tomich.^4^

Melanoacanthoma of the skin is a benign mixed proliferation of keratinocytes and melanocytes and is considered to be a variant of seborrheic keratosis. Most patients are adults, beyond 40 years of age. Sex predominance is not known.^5^ Most melanoacanthomas are located on the trunk, though lesions have been reported on the scalp, neck and extremities too.^5,6^ These lesions are almost exclusive to whites in middle to late age, developing slowly over a long period, and usually having a roughened or papillary surface.^7^

On the contrary, intraoral melanoacanthomas tend to affect a much younger population, occurring almost exclusively in blacks, with a female predilection. These lesions show rapid increase in size and may attain dimensions of several centimeters in a few weeks. Buccal mucosa is the most frequently reported intraoral site, although masticatory mucosa subject to chronic trauma (palate, gingiva) may also be affected.^8-11^ Involvement of labial mucosa^12-14^ and alveolar ridge^15^ has also been reported. Mostly unilateral and solitary,^16^ these deeply pigmented lesions may have a flat or slightly raised surface. The other end of the spectrum of clinical presentation includes lesions that may be bilateral,^17^ and even multifocal,^1,8,18,19^ as well as those which even have a proliferative or warty surface. These intraoral hypermelanotic macules or papules are typically brown, black or blue-black in color, with possible variation in the intensity of pigmentation.^1,3,18,20^

Intraoral melanoacanthoma still continues to be a rare entity.^21-24^ Some of the previously reported cases have been summarized in Table 1.

## CASE REPORT

A 12-year-old male patient presented for evaluation of a lesion in the left maxillary gingiva, which was present for the past 6 months. The patient was under medication with valproic acid for the treatment of petit mal seizures, till the age of 8 years, after which it was discontinued. Otherwise, the medical history was noncontributory.

**Fig. 1:**
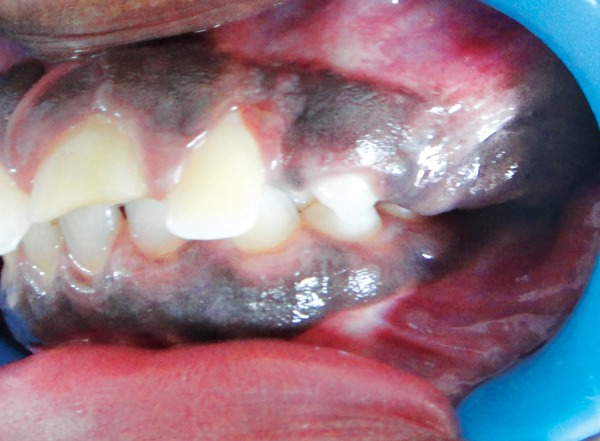
Clinical photograph showing the melanotic gingival enlargement in the maxillary left quadrant

**Fig. 2:**
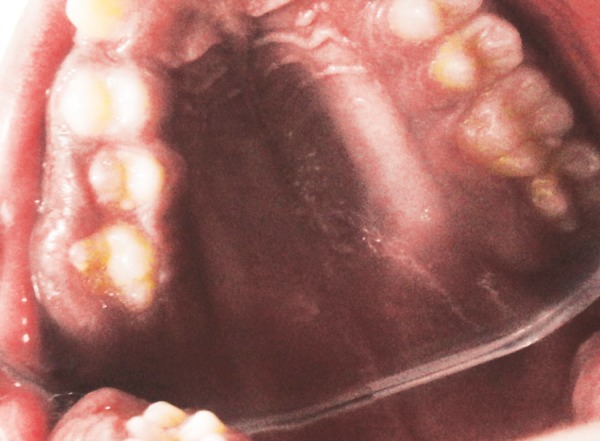
The lesion covering the entire buccal surfaces of the teeth and also partially covering the occlusal surface of the first molar

Extraoral examination revealed no clinically significant findings. Intraorally, there was a soft tissue growth in maxillary left quadrant (Fig. 1), involving the attached and the marginal gingiva on the buccal aspect. The lesion was brownish black in color and had a smooth, slightly raised surface (Fig. 2). The patient denied any association of pain with the lesion. The other three quadrants showed macular pigmentation of the attached and marginal gingivae, which was clinically labeled as racial pigmentation.

**Table 1:** **Table 1:** Summary of previously reported cases

*Authors*	*Years*	*Number of cases*	*Affected oral site*
Tomich^4^	1978	1	Buccal mucosa
Matsouka et al^14^	1979	1	Labial mucosa
Schneider et al^22^	1981	1	Buccal mucosa
Wright et al^20^	1983	2	Buccal mucosa
Goode et al^15^	1983	10	Buccal mucosa, palate, labial mucosa, alveolar ridge, attached gingiva
Frey et al^23^	1984	1	Buccal mucosa
Sexton and Maize^13^	1987	3	Labial mucosa
Wright^3^	1988	1	Buccal mucosa
Whitt et al^24^	1988	1	Buccal mucosa
Horlick et al^21^	1988	2	Buccal mucosa
Heine et al^17^	1996	1	Buccal mucosa
Chandler et al^1^	1997	1	Palate, tonsillar fossae, upper nasopharynx
Landwehr et al^16^	1997	1	Buccal mucosa
Flaitz^11^	2000	1	Attached gingiva
Fatahzadeh et al^18^	2002	1	Buccal mucosa, palate
Fornatora et al^9^	2003	10	Buccal mucosa, gingiva, hard palate, lower lip, floor of mouth, retromolar pad
Kauzman et al^19^	2004	1	Buccal mucosa, labial mucosa, tonsillar pillars
Carlos-Bregni et al^10^	2007	4	Gingiva, buccal mucosa, hard palate
Marocchio et al^8^	2009	1	Buccal mucosa, lips, gingiva, tongue

**Fig. 3:**
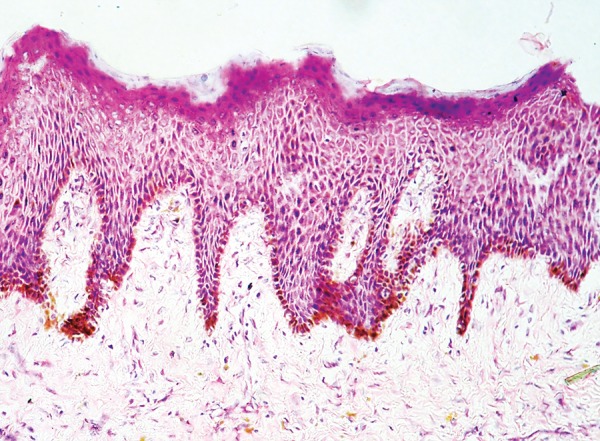
Photomicrograph showing acanthosis and parakeratosis of the surface epithelium as well as linear melanocytic hyperplasia in the basal layer. Benign melanocytes are seen in parabasal layers, and there is evidence of melanophagic activity

**Fig. 4:**
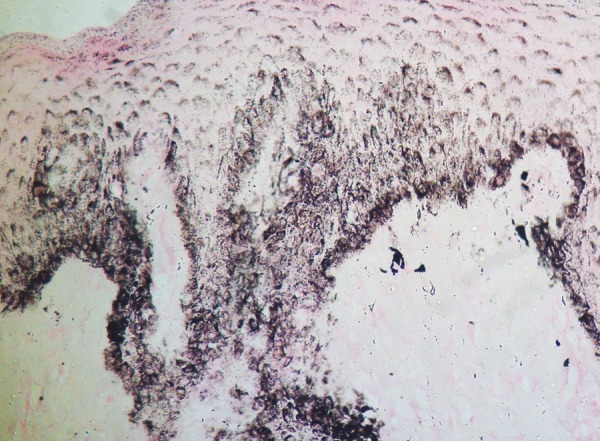
Photomicrograph highlighting the dendritic morphology of the melanocytes, which are seen extending above the basal layers (Masson-Fontana, 400×)

The lesion was excised and sent for histopathological examination. Hematoxylin and eosin (H&E) stained sections revealed surface stratified squamous epithelium and underlying fibrous connective tissue. The epithelium exhibited parakeratosis and acanthosis and the rete ridges were irregular in shape. There was a prominence of melanocytes in the basal layer, in a linear fashion (Fig. 3). There was a suspicion of pigmented melanocytes even in the suprabasal layers. The underlying connective tissue appeared normal, showing some evidence of melanophagic activity in the subepithelial zone. Masson-Fontana silver stain supplemented the presence of dendritic melanocytes filling up almost the entire epithelium (Fig. 4). The presence of benign appearing melanocytes was salient, and there was no evidence of any cytological atypia, pleomorphism or nuclear hyperchromasia. In light of the history, clinical features and the histopathological picture with H&E and Masson-Fontana stain, the final diagnosis of oral melanoacanthoma was rendered.

**Fig. 5:**
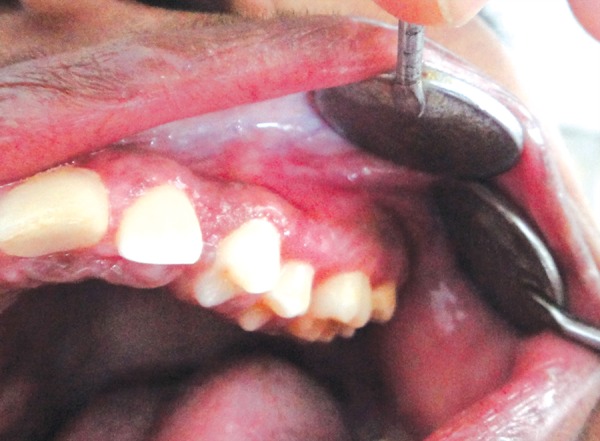
Postoperative clinical appearance of the site after 4 weeks

The patient has been on a regular follow-up (Fig. 5), and the lesion was observed to be healing well 10 months postoperatively.

## DISCUSSION

The credit for the first fully documented case of oral melanoacanthoma goes to Matsouka (1979).^14^ Since then, there has been an addition of more than 65 cases to the available literature. The pathogenesis still remains obscure, though some authors have ascribed the potential role of chronic trauma in these cases.

The intraoral melanoacanthoma is essentially reactive in nature'a fact supported by the clinical course of the lesion. It is characterized by a tendency to affect the mucosal sites that are exposed to trauma, and typically shows rapid growth and observable regression of the lesion'spontaneously or following incomplete removal^15^ or elimination of local irritants.^18^ The histologic picture of subepithelial inflammatory cell infiltrate and slightly increased vascularity further add evidence to this concept. To differentiate these lesions from cutaneous melanoacanthoma and to emphasize their reactive nature, several terms have been suggested, including melanoacanthosis, reactive melanocytic hyperplasia and mucosal melanotic macule.^6,8,21^

The clinical picture of this lesion is indistinguishable from many other oral pigmented lesions. All pigmented lesions should be observed for evolution with respect to size, shape, color, surface or symptom overtime.^2^ The observation of any of these features mandates a biopsy, because of potential resemblance to a malignant melanoma. The alarming growth rate of oral melanoacanthoma makes it clinically indistinguishable from oral malignant melanoma, especially the radial growth phase of an *in situ* melanoma. The biopsy, hence, should be performed invariably to rule out the possibility of a melanoma. Once the diagnosis has been established, no further treatment is indicated, and many cases document spontaneous regression.

The diagnosis of oral melanoacanthoma can be made solely on the basis of histological features and special staining. In order to emphasize the presence of melanin and to demonstrate the dendritic melanocytes, Masson-Fontana silver impregnation technique can be used.^10^ The immunohistochemical profile of these lesions is limited to the melanocytic markers, but is not necessary for diagnosis, as strong reactivity to HMB-45 and S100 is seen in both oral melanoacanthoma and malignant melanoma.^8-10^

Some authors^6,8^ opine that in contrast to the other pigmented lesions, the melanin in oral melanoacanthoma is restricted mainly to melanocytes, the adjacent keratinocytes being devoid of melanin. Interestingly, in our case, the histopathological picture showed the presence of ‘dusty’ melanin in the basal as well as parabasal keratinocytes.

Melanoacanthoma is a reparative lesion with no malignant potential. The treatment should be directed toward removing all local causes of trauma and excluding any other causes of oral pigmentation, particularly malignant melanoma.

The authors advocate the replacement of the misnomer ‘melanoacanthoma’ with a more appropriate description ‘melanoacanthosis', a term which gives due credit to the clinical behavior and histopathological picture of this rare and interesting lesion.

## SUMMARY

We present a case of a rare entity, oral melanoacanthoma, occurring at a rarer oral mucosal site, that is, gingiva, in a 12-year-old Asian male. The diagnosis was based mainly on the histologic findings with H&E and Masson-Fontana stains. The patient is on a regular follow-up and the lesion has regressed completely after the initial surgery.
